# Inclusion and Exclusion Criteria of Clinical Trials for Insomnia

**DOI:** 10.3390/jcm7080206

**Published:** 2018-08-08

**Authors:** Hendrikje Huls, Smedra Abdulahad, Marlou Mackus, Aurora J. A. E. van de Loo, Timothy Roehrs, Thomas Roth, Joris C. Verster

**Affiliations:** 1 Division of Pharmacology, Utrecht University, 3584CG Utrecht, The Netherlands; h.huls@students.uu.nl (H.H.); s.abdulahad@students.uu.nl (S.A.); m.mackus@uu.nl (M.M.); a.j.a.e.vandeloo@uu.nl (A.J.A.E.v.d.L.); 2 Institute for risk assessment Sciences (IRAS), Utrecht University, 3584CM Utrecht, The Netherlands; 3 Sleep Disorders & Research Centre, Henry Ford Hospital, Detroit, MI 48202, USA; troehrs1@hfhs.org (T.R.); troth1@hfhs.org (T.R.); 4 Centre for Human Psychopharmacology, Swinburne University, Melbourne, VIC 3122, Australia

**Keywords:** clinical trial, efficacy, safety, insomnia, eligibility, recruitment, screening, inclusion criteria, exclusion criteria, patient selection

## Abstract

Randomized controlled trials (RCTs) have eligibility criteria for the inclusion of participants. Ideally, the RCT sample would be representative for the patient population that will use the drug under investigation. However, external validity may be at stake when applying too many or too restrictive eligibility criteria. The current two-part study examined (1) the currently applied eligibility criteria in Phase II and III RCTs examining sleep medication; (2) how these criteria match with the insomnia population as a whole; and (3) how inclusion rates can be changed by an adaptation of these criteria. In the first study, insomnia RCTs were screened at www.clinicaltrials.gov, and relevant eligibility criteria were identified. The second study comprised a survey among self-reported insomnia patients. It was determined to what extent RCT eligibility criteria match the characteristics of this patient population. Of the *n* = 519 patients that completed the survey only *n* = 2 (0.4%) met all eligibility criteria of current RCTs. RCT enrolment criteria are not representative for the insomnia patient population as a whole. Being less rigorous in applying upper or lower criteria limits results in a significant increase in the number of eligible patients, and increases the representativeness of RCTs for the insomnia patient population as a whole. The current analysis demonstrates that is important to thoroughly reconsider the use eligibility criteria and their inclusion ranges, and to have a theoretical basis for using them.

## 1. Introduction

Research of the safety and efficacy of new medicinal drugs is usually conducted via randomized controlled trials (RCTs) [1]. An important part of RCTs is the selection of eligible patients. However, the number and type of in- and exclusion criteria vary greatly between RCTs. While some RCTs allow for a broad sampling of patients, other RCTs use a very restricted profile for patient eligibility. Importantly, selection criteria should not exclude groups without valid reasons, as this negatively affects the external validity of the RCT [2]. 

Enrolment criteria were originally designed to recruit patients that were representative of the individuals who will ultimately use the medication clinically. For instance, patients were required to suffer from the disease that is being treated by the newly developed drug, and patients should not have confounding factors, such as co-medication or comorbidity, which make the study outcomes more difficult to interpret. More importantly, an individual should not be put at unnecessary risk by participating in an RCT. For example, patients with an allergic reaction to the drug under investigation, or with a risk for potential worsening of a concurrent disease, are usually excluded from participation. Similarly, women are evaluated for pregnancy, and adequate birth control measures are required for inclusion in an RCT. 

Insomnia is characterized by difficulty falling asleep, maintaining sleep, or both, and is reported by 10% to 20% of the US population [3]. Similar prevalence rates are reported for China [4] and Europe [5]. A recent study examined the recruitment process of two RCTs in insomnia [6]. Individuals who passed the telephone interview were screened at the clinic. Of those, 18% of the US and 38% of Dutch samples did not meet additional insomnia criteria, another 28% of the US and 32% of Dutch samples were excluded for mental health reasons, medical health problems accounted for additional exclusion of 21% of US and 9% of Dutch patients, and 16% of the US and 26% of Dutch samples were excluded for drug use/abuse histories. As a result, only 4% of the initial US contacts and 0% of the Dutch patients entered the RCT. It was concluded that the data suggest that persons who are eligible to participate in insomnia clinical trials comprise a highly-selected sample that is unlikely to be representative of the insomnia population as a whole. The very small number of eligible patients is a direct result of having many strict in- and exclusion criteria. It can be questioned whether these eligibility criteria are all equally important, and whether the chosen upper and lower limits for screening assessments (e.g., BMI ranges) are critical for safety or efficacy reasons, or based on consensus of what would seem an acceptable normal range.

Van Spall et al. suggested that enrolment criteria of RCTs can be divided into three categories, which reflect the justification for using them [7]. These categories are ‘strongly justified reasons’, ‘potentially justified reasons’, and ‘poorly justified reasons’. Strongly justified reasons are exclusion criteria that prevent a drug intervention from being harmful or irrelevant. Potentially justified reasons are criteria that are related to adherence to the intervention or treatment compliance. Poorly justified reasons exclude participants for unclear reasons that are not obviously linked to the disease, adverse events, or the potential outcome of the intervention. For strongly justified reasons a specific rationale can be provided, whereas this is less or not the case for potentially and poorly justified reasons, respectively. 

This categorization can be applied to eligibility criteria of all RCTs, independent of the disease under investigation. In the case of insomnia, examples of strongly justified criteria could be that the participant’s insomnia complaints are defined by several factors, such as sleep duration, sleep latency, and maintenance and bed time. Examples of poorly justified criteria could be those that exclude participants who, in the past, suffered from a disease which is currently resolved, or occasional past drug use (e.g., a 45 year old drug-free potential participant who acknowledges occasionally smoking marijuana when he was 21 years old). It is difficult to defend that these criteria will have an impact on the RCT outcome. According to the National Institutes of Health (NIH), RCT eligibility criteria must be clear, practical, permitting generalizability, and establish the ethical foundation of the study [8]. If eligibility criteria are too specific, there is a risk of the research not being representative for the population of potential drug users. This selection bias can cause unexpected events when a drug is brought on the market and will be used by the insomnia population at large that does not meet the strict RCT criteria. When a drug is not tested within a representative sample, there is no certainty with regard to drug efficacy and safety in these groups. Post marketing data can identify potential risk factors, but it would be more ideal to identify these during drug development. For this reason, RCT selection criteria must be cautiously chosen and strongly justified. 

Given the sometimes arbitrary use of inclusion and exclusion criteria without a good theoretical basis, it is important to investigate to what extent these criteria represent a patient population, how the specific criteria influence eligibility rates in RCTs, and how inclusion rates can be changed by an adaptation of these criteria. The current study, therefore, aimed to summarize the enrolment criteria of all recent RCTs in insomnia listed at www.clinicaltrials.gov, and critically review the validity of these inclusion and exclusion criteria. Subsequently, self-reported patients with sleep initiation and maintenance problems (insomnia) were invited to complete an online survey, to determine how many of them met the various eligibility criteria of current RCTs in insomnia. Finally, it was verified to what extent being more flexible with inclusion ranges of eligibility criteria has a positive impact on the inclusion rates of RCTs.

## 2. Experimental Section

### 2.1. Eligibility Criteria Selection

**Figure 1:**
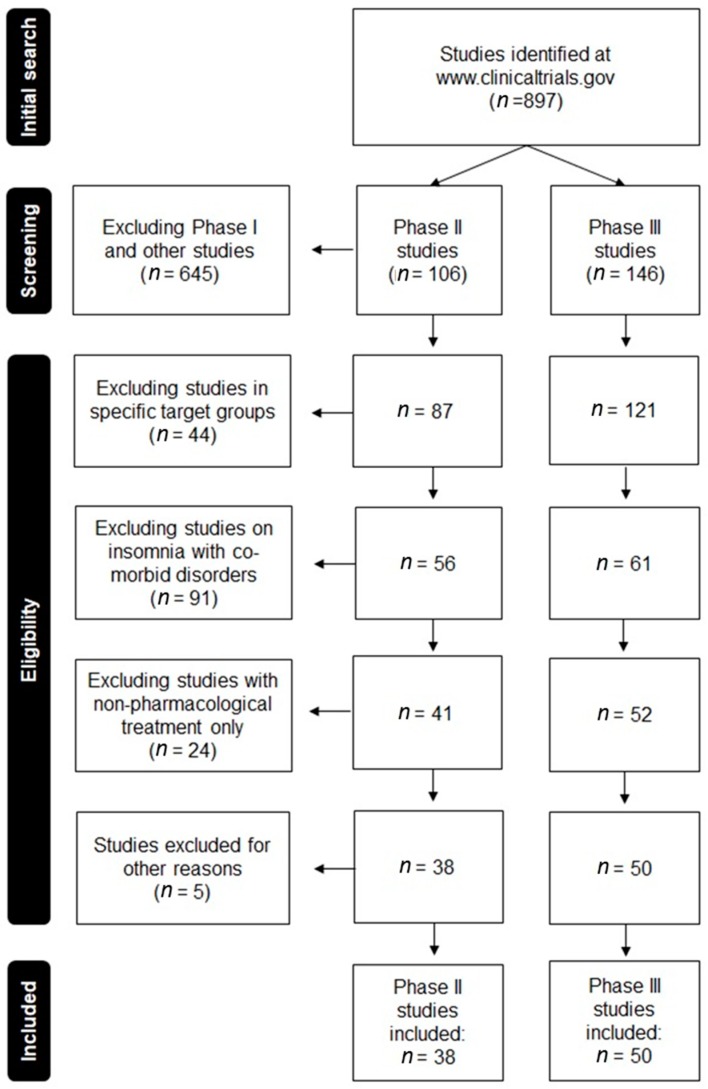
Flow chart summarizing the selection of relevant Phase II and III clinical trials in insomnia.

Data from RCTs was extracted from www.clinicaltrials.gov, on February 28, 2018. An overview of the study selection process is given in Figure 1. The initial search conducted using the search term ‘insomnia’, which resulted in 897 RCTs. Selecting for Phase II studies identified 106 RCTs and selecting for Phase III studies identified 146 RCTs. These studies were reviewed for the following criteria: (1) the sample should consist of insomnia patients (not healthy volunteers); (2) the intervention must be a pharmacological treatment developed to treat insomnia (RCTs examining behavioral treatments of lifestyle interventions were excluded). Applying this selection, 38 Phase II and 50 Phase III RCTs were included in the analysis (see Figure 1).

Of the 88 RCTs that remained after the selection process, the inclusion and exclusion criteria were extracted and analyzed. First, some criteria were grouped together as they measure the same concept but used different terminologies. For sleep onset latency, two criteria were used (30 or 45 min), as they are both often used. Second, criteria that were used for both inclusion and exclusion were combined and allocated to one of these categories. The nature and extent of eligibility criteria was assessed using the classification proposed by Van Spall et al. [7]. Accordingly, criteria were labelled as poorly, potentially or strongly justified. The eligibility criteria that were applied in the 88 RCTs (although their frequency of use varied) are listed in Table 1.

### 2.2. Online Survey

Subjects (aged 18 and older) with sleep problems (either diagnosed or not) were invited via an advertisement at www.facebook.com to participate in an online survey. The advertisement stated that both men and women, 18 years and older, were asked to participate if they experience difficulty initiating and/or maintaining sleep. The survey was designed using www.surveymonkey.com. Informed consent was obtained from all participants, and the Ethics Committee of the Faculty of Social and Behavioural Sciences of Utrecht University approved the study.

The survey that contained questions about demographics, lifestyle, health status, sleep habits, and medication use, and was designed to gather information on several of the RCT eligibility criteria summarized in Table 1. Eligibility criteria that could not be measured via a survey, for example, because medical devices are needed to conduct clinical assessments (e.g., blood pressure), and are indicated in Table 1 by *. No assessment of insomnia severity was included, as this criterion was mentioned only in a few RCTs, and different assessment methods were used. Finally, eligibility criteria related to compliance and scheduling of appointments, or lifestyle adaptations specific for the RCT (e.g., a willingness to adhere to a fixed bedtime) were also not included in the survey (see Table 1). 

### 2.3. Statistical Analysis

Statistical analysis was conducted using SPSS, version 24 (IBM, Armonk, NY, USA). Body mass index (BMI) was calculated by dividing weight (kg) by squared length (m). Total caffeine consumption per day was calculated using caffeine content and proportion sizes as used by Mackus et al. [9]. Total sleep time (TST) was calculated by subtracting sleep-time from wake-time and sleep onset latency (SOL) by subtracting lights-out time from falling asleep-time. 

In the first analysis, for each eligibility criterion the percentage of subjects that met the criterion was computed. Descriptive statistics (frequency counts) determined the percentage of participants being included or excluded (i.e., met the criterion) based on the criteria summarized in Table 1.

Second, a stepwise analysis was conducted to show how many subjects could be included if *n* + 1 criteria should be met, starting with the criterion that was found most frequently mentioned in relevant RCTs identified at www.clinicaltrials.gov (Part 1, see Table 1). 

In the third analysis, the percentage of eligible subjects was determined when including all criteria, but omitting one specific criterion per analysis. The relative impact of the criterion was computed by subtracting the percentage of eligible patients when all criteria were included (0.4%) from the percentage of eligible patients when a specific criterion was omitted.

Fourth, an analysis was conducted in which either all poorly, potentially, or strongly justified criteria were omitted. This analysis provided insight into the relative influence of these three criterion categories. 

Finally, the distributions of selected sleep-related strongly justified criteria (BMI, TST, SOL, habitual bedtime) were plotted to investigate the effect of increasing or reducing the upper or lower limit on eligibility rates. With this analysis it could be determined what the impact of changing these eligibility criteria (e.g., raising the BMI upper limit from 32 to 33 kg/m^2^) would have on the percentage of subjects that falls within the set limits for the criterion (i.e., would be considered eligible to participate in an RCT).

## 3. Results

*n* = 1031 subjects started the survey. *n* = 25 were excluded as they did not consent to perform the survey, *n* = 94 were excluded due the data being unreliable, and another *n* = 41 were excluded because they reported not to have any sleep problems. *n* = 871 subjects, of which *n* = 698 (83.1%) were women, were included in the statistical analysis. Their mean (SD) age was 37.0 (13.3), and they had a mean (SD) BMI of 26.6 (5.6). In the first analysis, for each criterion the percentage of subjects that met the criterion was computed. Table 2 gives an overview of the results.

The percentage of subjects excluded differs greatly between various criteria. The highest number of participants were excluded based on not being formally diagnosed with insomnia or women not using contraception. Factors that hardly had any impact were those that concerned medical history (cancer, drug or alcohol abuse, neurological disorder), pregnancy, age, and alcohol intake. The highest percentages are found in the potentially justified criteria. The poorly justified criteria show great heterogeneity, as do the strongly justified criteria to some extent. 

In the second analysis, a stepwise analysis was conducted to show how many subjects could be included if *n* + 1 criteria should be met, starting with the criterion that was most frequently mentioned in relevant RCTs identified at www.clinicaltrials.gov (Part 1, see Table 1). The data is presented in Table 3. The analysis revealed that only two participants out of 519 (0.4%) would meet all inclusion and exclusion criteria.

In the third analysis, the variability in percentage of eligible patients was determined when including all criteria, but omitting one criterion per analysis. This analysis enables to identify the impact of individual inclusion and exclusion criteria on the overall eligibility rate. All criteria listed in Table 3 were used for the analysis. Criteria that were not included in Table 4 had no impact on the overall eligibility rate. The analysis revealed that omitting individual eligibility criteria has no relevant effect on the overall eligibility rate (see Table 4).

The fourth analysis showed that the three classes of criteria and their combinations show large differences on eligibility rates (see Table 5). Poorly justified criteria exclude the fewest subjects (67.1%) while the strongly (89.8%) and potentially justified criteria (95.0%) exclude much more subjects. If all strongly and potentially justified criteria should be met, only three participants (0.6%) of the survey sample would meet all inclusion and exclusion criteria. When only strongly justified criteria should be met, 10.2% would meet all inclusion and exclusion criteria. 

**Figure 2:**
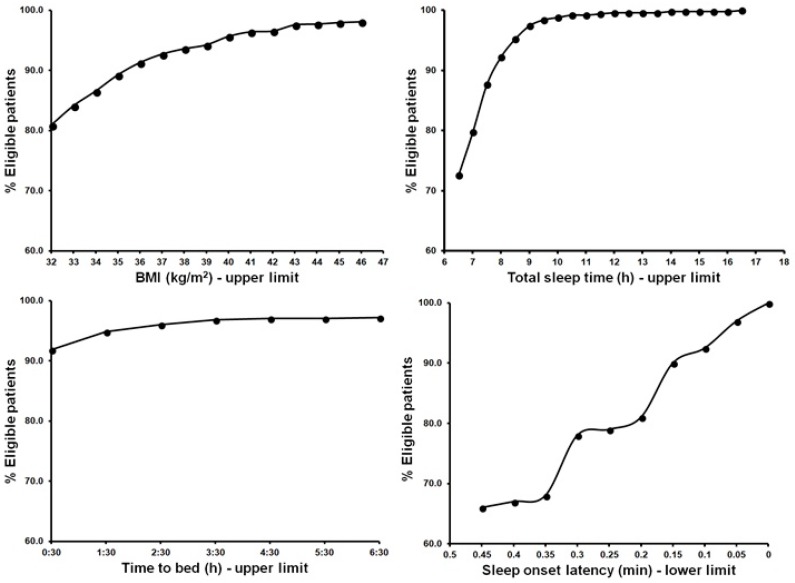
Percentage of eligible patients as an effect of changing an upper or lower criterion limit. BMI = body mass index.

Finally, analysis of the flexibility of upper limits of some eligibility criteria revealed that increasing the limits has a significant effect on the number of participants that would meet the criterion. Figure 2 illustrates this effect for BMI, TST, habitual bedtime, and SOL. It is evident from Figure 2 that being flexible with the set limits for specific criteria can have a significant impact on eligibility rates. For example, increasing the upper BMI limit from 32 kg/m^2^ to 33 kg/m^2^ results in an increase of 4% of patients meeting the BMI criterion. Alternatively, reducing the SOL criterion from 45 min to 30 min results in an increase of 12% of patients meeting the SOL criterion.

## 4. Discussion

The findings of this study suggest that randomized clinical trials for insomnia are not representative for the general patient population who would potentially take this type of medication. Only two out of 519 potential participants (0.4%) met all inclusion and exclusion criteria that are currently used in RCTs. These findings are in line with a recent study investigating the eligibility rates of insomnia patients for two RCTs conducted in USA and The Netherlands, in which after screening only 4% of the initial US insomnia patients and 0% of the Dutch insomnia patients were eligible for participating in the corresponding RCT [4].

The eligibility criteria that most frequently led to exclusion were having no formal diagnosis of insomnia, not using a medically approved contraceptive method, ongoing use of (sleep) medication, comorbidity, time spent in bed, and tobacco use. These eligibility criteria were used in the majority of RCTs (18.2% to 63.6% of the studies identified at www.clinicaltrials.gov). 

RCTs that examined other disease areas, such as obsessive-compulsive disorder and heart failure, show comparable results [10,11]. Geller et al. found that psychiatric comorbidity negatively impacted the pharmacotherapeutic response and concluded that the majority of RCTs done in this area of research excluded too many patients [11]. Heiat et al. argued that minorities, elderly and women are frequently excluded from RCTs and that the clinical impact of this underrepresentation is unknown [10]. Women are often excluded because they not meet the criterion of using specific contraception. In our survey, 52.5% of women did not meet this criterion. Whereas in the 20th century, all women with childbearing potential were excluded from participating in RCTs because of potential risks for the foetus, this is no longer viewed as an acceptable exclusion criterion, per se, as it excludes many women [12]. Although this criterion was classified as potentially justified, this does not imply that women should be excluded simply because they do not use pharmacological or device contraception methods. For instance, a woman not willing to take contraception, may be using other precautions like abstinence or have a non-fertile sexual partner. Unfortunately, in RCTs these methods are often not regarded as acceptable preventive measures.

Many of the exclusion rates reported by van Spall et al. [7] were comparable to the current findings: in both studies common medical disorders, age, medication, and conditions related to the female sex (e.g., contraceptive methods, breast feeding) were all the basis for exclusion in at least 50% of all clinical trials in van Spall’s analysis. 

Van Spall et al. [7] classified having comorbid disorders as a poorly justified criterion, as it comprises a very large, heterogeneous group of patients, of which it is usually not defined to what extend specific diseases do or do not have a potential impact on the study outcome. The exclusion of patients with comorbid diseases in common in many RCTs (84.1% of studies identified at www.clinicaltrials.gov). As insomnia is often accompanied by comorbidities [13,14], excluding these patients may greatly reduce the representativeness of the eligible sample for the insomnia population as a whole. In our survey, 39% of all subjects reported having a clinically significant comorbid disorder that would exclude them from participation. 

Elderly patients are often excluded from RCTs, despite several regulatory agencies urging to include them [15]. Elderly were also excluded in 75% of the selected RCTs at www.clinicaltrials.gov. The percentage elderly with insomnia in the general population is higher than the numbers we observed in our survey (2.1%). The latter is likely due to the fact that only Facebook was used to invite participants to complete the survey, and it is known that, compared to younger adults, the elderly are less likely to use social media (i.e., only 4% of active Facebook users are 65 years old or older) [16]. As insomnia is more common in the elderly [17], they should be included in future clinical trials. 

Omitting individual criteria has little impact on overall the inclusion rate (see Table 4). Applying van Spall’s classification [7], it appeared that most influential criteria that led to exclusion are strongly justified criteria. When only applying strongly justified criteria, the eligibility rate of the survey sample would increase by 10.2% (see Table 5). The most frequently not met inclusion criterion observed in the survey was being formally diagnosed for having insomnia. Three out of four participants (75.4%) were excluded because they did not meet this inclusion criterion. This criterion was classified as potentially justified. Although it is important that the patient suffers from the disease under investigation, the lack of a formal diagnosis should not exclude potential participants upfront, as they can also be diagnosed by the study physician as part of the screening process of an RCT. 

BMI and tobacco use are common exclusion criteria, but both are classified as poorly justified criteria. BMI criteria were found in 42% of the RCTs identified at www.clinicaltrials.gov, and excluded 19.1% of the potential participants of our survey sample. A tobacco use criterion was set in 25% of the RCTs identified at www.clinicaltrials.gov, and in the survey 34.8% of the participants did not meet this criterion. Van Spall et al. classified the criteria BMI and tobacco use as poorly justified, as no adverse drug effects have been associated with them, and usually no justification is given for applying these exclusion criteria [7]. Alcohol use is also a frequently used exclusion criteria in RCTs (23.9% of the RCTs identified at www.clinicaltrials.gov). In the survey, only 1.9% of the potential participants did not meet the alcohol consumption criterion. Eligibility criteria concerning smoking and alcohol consumption are set in many RCTs, also outside the field of insomnia, presumably because these habits may affect general health. However, a specific justification of applying these criteria in terms of affecting the efficacy or safety of the drug under investigation is usually not provided. 

One could argue that being more flexible with inclusion and exclusion criteria is necessary to increase the match between the eligible subject sample of an RCT and the future patient population that will use the medication under investigation. Figure 2 illustrates the effect of being more flexible with the eligibility criteria limits for BMI, TST, and sleep onset latency. Interestingly, relatively small changes of the upper and lower limits of these criteria can generate a substantial increase in eligible patients. For example, raising the BMI upper limit from 32 kg/m^2^ to 33 kg/m^2^ increases the percentage of eligible patients by almost 4%. As the BMI is classified as a poorly justified criterion, one could consider being more flexible about its inclusion range. 

The other three criteria were classified as strongly justified and are, thus, important criteria to be used for participant selection. However, for these criteria one could also determine whether it is feasible to be less rigorous regarding upper or lower limits of inclusion. For example, in our survey sample a reduction of SOL lower limit from 45 min to 30 min, two lower limits that are commonly applied in various studies, results in 12% more eligible patients. Thus, being more flexible on eligibility criteria, without sacrificing safety or affecting drug efficacy, will significantly increase the number of eligible participants for an RCT and, more importantly, this will also increase the external validity of a trial. 

### Limitations

There are some limitations to the present study that, albeit they had limited effect on the results, should be addressed. Firstly, the data from the insomnia patients was gathered via an online survey distributed via a Facebook advertisement. Although this generated a large number of potential respondents in a short amount of time, some parts of the population are underrepresented on Facebook [16]. As mentioned earlier, the elderly are much less active on social media and, thus, were less effectively approached to participate in the survey. Additionally, significantly more women responded to the survey than men (83.1%). Although insomnia is more frequently seen in women [18], the reported difference is usually not that large. One could speculate that the observed sex difference in respondents is due to women being more likely to complete online surveys, being more active on social media, or being more attracted by the topic of the survey than men. 

Furthermore, not all eligibility criteria summarized in Table 1 could be examined in the survey. Those include criteria that require medical devices to assess them, but also more practical criteria, such as availability for appointment scheduling and willingness to adhere to certain lifestyles, which are less straightforward to investigate using a survey design. However, even without including these eligibility criteria in the survey, almost nobody of the survey sample met all eligible criteria for participating in an RCT. In retrospective, in future studies it would also be interesting to include a measure of insomnia severity to better characterize the RCT sample and help to (re)-diagnose insomnia. However, consensus should be reached about the specific assessment scale should be reached, and its cut-off for screening positive for insomnia. Additionally, some other criteria that were currently not included (e.g., a question on napping was unintentionally omitted) could be considered in future research. In future research it would also be interesting to also examine specific populations (e.g., shift workers), or RCTs examining non-pharmacological treatments such as behavioural treatments of lifestyle interventions Finally, common limitations of survey research include the possibility of obtaining inaccurate answers due to recall bias or socially desirable answering. While the latter is unlikely to have had an impact as we conducted an anonymous survey, recall bias can always have impacted self-report. Also, some questions required answers that are more difficult to provide accurate by self-report than others (e.g., sleep onset latency versus the use of contraception).

## 5. Conclusions

One of the aims of the current paper was to open the discussion among sleep specialists and RCT designers about the implications of applying specific eligibility criteria, to thoroughly think about these criteria, and to have a theoretical basis for using them. However, research into enrolment criteria is very limited and the consequences of proposed eligibility criteria for inclusion rates of RCTs are often not appreciated by people who design RCTs. This study demonstrated that not only the number of eligibility criteria used in insomnia RCTs has a significant impact on inclusion rates, but also that their relatively small range for inclusion (e.g., upper BMI limits) has a relevant impact on inclusion rates. However, being more selective in the use of eligibility criteria and more flexible in the applied inclusion ranges is a cost-effective and time-reducing strategy to increase the number of eligible patients in a clinical trial. Given that inclusion ranges are often set arbitrary or by consensus, there should be room for debate for applying broader inclusion ranges, especially when these reflect the overall insomnia population. Together, the current observations raise concerns about the recruitment strategy of RCTs and the generalizability of their results: if only 0.4% of patients that consider themselves to have insomnia meet all criteria to be included in an RCT, it can be questioned how representative the outcomes with regards to drug efficacy and safety are for the insomnia population as a whole. 

## Figures and Tables

**Table 1:** Inclusion and exclusion criteria.

Frequency	Criterion	Type and Range	Justification
69	Pregnant or lactating (females)	Exclusion if yes	Strong
40	Nightshift or rotating shift work	Exclusion if yes	Strong
32	Sleep disorder other than insomnia	Exclusion if yes	Strong
28	Medication affecting sleep	Exclusion if yes	Strong
24	Total sleep time (h)	Inclusion if <6.5	Strong
23	Sleep onset latency (min)	Inclusion if >30 or >45	Strong
19	Time in bed (h)	Inclusion if 6.5–9	Strong
18	Use of caffeine (mg)	Exclusion if >600	Strong
16	Habitual bedtime (h)	Inclusion if 21:00–00:30	Strong
15	Wake after sleep onset (min) *	Inclusion if >60	Strong
14	Number of naps per week *	Inclusion if >3	Strong
8	Apnoea-hypopnea index *	Exclusion if >10	Strong
7	Periodic leg movement with arousal index *	Exclusion if >10	Strong
7	Insomnia severity index *	Inclusion if >14	Strong
7	Willing to have a fixed bedtime, remain in bed for 8 h *	Inclusion if yes	Strong
6	Willing to comply with RCT restrictions and clinic visits *	Inclusion if yes	Strong
6	Willing to complete surveys at home, access to phone *	Inclusion if yes	Strong
4	Positive urine drug screen *	Exclusion if yes	Strong
2	Pittsburgh Sleep Quality Index *	Inclusion if >4	Strong
1	Sleep efficiency (%) *	Inclusion if <85	Strong
2	Positive alcohol breathalyser test *	Exclusion if yes	Strong
1	Multivariable Apnoea risk index *	Exclusion if >0.5	Strong
1	Epworth Sleepiness Scale score *	Inclusion if <9	Strong
56	Diagnosed with primary insomnia	Inclusion if yes	Potential
38	History of alcohol or drug abuse	Exclusion if yes	Potential
27	Use of prescription drugs or clinically significant drugs	Exclusion if yes	Potential
21	Alcohol consumption (units per day)	Exclusion if >2	Potential
16	Use of contraception (pre-menopausal females)	Exclusion if no	Potential
3	Haematology deviating from normal range *	Exclusion if yes	Potential
2	Creatine clearance (mL/min) *	Exclusion if <30	Potential
2	AST/ALT (UNL) *	Exclusion if >2	Potential
1	Bilirubin (UNL) *	Exclusion if >1.5	Potential
1	Not euthyroid as evident by normal TSH *	Exclusion if yes	Potential
1	Glomerular filtration rate (mL/min) *	Exclusion if <30	Potential
74	Clinically significant psychiatric, neurological, or medical disorders	Exclusion if yes	Poor
60	Age (years)	Inclusion if 18–65	Poor
37	Body mass index (kg/m^2^)	Inclusion if 18–32	Poor
24	History of significant neurological disorder	Exclusion if yes	Poor
23	Tobacco use	Exclusion if yes	Poor
19	History of sleep disorder other than insomnia	Exclusion if yes	Poor
6	ECG parameters outside of specified range *	Exclusion if yes	Poor
5	Outpatient *	Inclusion if yes	Poor
4	History of cancer	Exclusion if yes	Poor
3	Systolic blood pressure (mm Hg) *	Exclusion if >150	Poor
3	Heart rate (bpm) *	Exclusion if >100	Poor
2	QT interval (msec) *	Exclusion if >450	Poor
1	2nd or 3rd degree atrioventricular block *	Exclusion if yes	Poor

AST = aspartate transaminase, ALT = alanine transaminase, ECG = electrocardiogram, TSH = thyroid stimulating hormone. * Not included in the survey.

**Table 2:** Eligibility rates of survey participants.

Criterion	Justification	% Excluded
Medication affecting sleep	Strong	43.8
Time in bed	Strong	39.4
Sleep onset latency (45 min)	Strong	34.1
Total sleep time	Strong	27.4
Sleep onset latency (30 min)	Strong	22.2
Nightshift or rotating shift work	Strong	18.1
Sleep disorder other than insomnia	Strong	8.9
Habitual bedtime	Strong	8.1
Use of caffeine	Strong	5.0
Pregnant or lactating (females)	Strong	1.4
Diagnosed with primary insomnia	Potential	75.4
Use of contraception (pre-menopausal females)	Potential	52.5
Use of prescription drugs or clinically significant drugs	Potential	40.6
Alcohol intake	Potential	1.9
History of alcohol or drug abuse	Potential	3.0
Clinically significant psychiatric, neurological, or medical disorders	Poor	39.0
Tobacco use	Poor	34.8
BMI	Poor	19.1
History of cancer	Poor	3.5
Age	Poor	2.1
History of significant neurological disorder	Poor	1.3

Exclusion of patients based on the criteria summarized in Table 1.

**Table 3:** Percentage of eligible patients after applying subsequent eligibility criteria (*n* + 1).

Criterion	Frequency	Eligible (*n*)	Patients (%)
Clinically significant psychiatric, neurological, or medical disorders	74	309	59.5
Pregnant or lactating (females)	69	303	58.4
Age	60	298	57.4
Diagnosed with primary insomnia	56	69	13.3
Nightshift or rotating shift work	40	52	10.0
History of alcohol or drug abuse	38	48	9.2
BMI	37	39	7.5
Sleep disorder other than insomnia	32	36	6.9
Medication affecting sleep	28	10	1.9
Use of prescription or clinically significant drugs	27	8	1.5
Total sleep time	24	7	1.3
History of significant neurological disorder	24	7	1.3
Tobacco use	23	5	1.0
Sleep onset latency (30 min)	23	4	0.8
Sleep onset latency (45 min)	23	4	0.8
Alcohol intake	21	4	0.8
Time in bed	19	4	0.8
Use of caffeine	18	4	0.8
Use of contraception (pre-menopausal females)	16	2	0.4
Habitual bedtime	16	2	0.4
History of cancer	4	2	0.4

**Table 4:** Percentage of eligible patients when a specific criterion was not applied.

Criterion	% Eligible	Impact (%)
All criteria included	0.4	-
Diagnosed with primary insomnia	1.3	+0.9
Use of contraception (pre-menopausal females)	0.8	+0.4
Use of drugs that affect sleep	0.8	+0.4
Tobacco use	0.6	+0.2
Body mass index	0.6	+0.2

The relative impact of the criterion was computed by subtracting the percentage of eligible patients when all criteria were included (0.4%) from the percentage of eligible patients when a specific criterion was omitted.

**Table 5:** Patient eligibility rates per classification.

Criterion Justification	Included	Excluded
Strong	53 (10.2%)	466 (89.8%)
Potential	26 (5.0%)	493 (95.0%)
Poor	171 (32.9%)	348 (67.1%)
Strong + Potential	3 (0.6%)	516 (99.4%)
Poor + Potential	9 (1.7%)	510 (98.3%)
